# BEING AND STAYING ELIGIBLE FOR SERVICES: PROVIDERS’ VIEWS ON SUPPORTING OLDER ADULTS EXPERIENCING HOMELESSNESS

**DOI:** 10.1093/geroni/igae098.1109

**Published:** 2024-12-31

**Authors:** Morgan Cruz Erisman, Sarah Canham, Lena Rebecca Richardson, Rachel Weldrick, Atiya Mahmood, Christine Walsh, Tamara Sussman

**Affiliations:** University of Utah, Salt Lake City, Utah, United States; University of Utah, Salt Lake City, Utah, United States; Simon Fraser University, Vancouver, British Columbia, Canada; McMaster University, Hamilton, Ontario, Canada; Simon Fraser University, Vancouver, British Columbia, Canada; University of Calgary, Calgary, Alberta, Canada; McGill University, Montreal, Quebec, Canada

## Abstract

Older people experiencing homelessness (OPEH) are a marginalized population with limited access to shelter/housing supports. Amidst these limited resources, understanding program-specific (in)eligibility criteria, along with strategies to sustain tenancy in different shelter/housing settings, is crucial to supporting OPEH in their housing goals. This study examined providers’ understanding of housing (in)eligibility in different shelter/housing settings, factors that impact the criteria for staying eligible, and interventions and steps providers use in assisting OPEH to maintain their eligibility and move towards their housing goals. Semi-structured interviews were conducted with 15 providers from five shelter/housing organizations serving OPEH across three Canadian cities. These organizations represented a range of shelter/housing services for OPEH, including emergency shelters, temporary/transitional housing, and permanent supportive housing. We conducted a thematic analysis and organized findings into three themes: 1) Medical and behavioral factors that impact (in)eligibility of OPEH for shelter/housing services; 2) Challenges to maintaining eligibility due to medical and behavioral factors; and 3) Provider and organizational support mechanisms to sustain tenancy. Findings provide insight into who is (in)eligible for different shelter/housing programs catered to OPEH, along with different support mechanisms available to support OPEH in their housing goals. Findings highlight the supports and services needed by OPEH with medical and behavioral challenges to remain eligible in different shelter/housing settings and recommendations for how to increase and target services to OPEH with specific needs. Understanding medical and behavioral factors that impact the shelter/housing tenancy of OPEH provides an opportunity to foster client success and promote positive housing outcomes.

